# Moringa Leaves Prevent Hepatic Lipid Accumulation and Inflammation in Guinea Pigs by Reducing the Expression of Genes Involved in Lipid Metabolism

**DOI:** 10.3390/ijms18071330

**Published:** 2017-06-22

**Authors:** Manal Mused Almatrafi, Marcela Vergara-Jimenez, Ana Gabriela Murillo, Gregory H. Norris, Christopher N. Blesso, Maria Luz Fernandez

**Affiliations:** 1 Department of Nutritional Sciences, University of Connecticut, Storrs, CT 06269, USA; manal.almatrafi@uconn.edu (M.M.A.); ana.murillo_solis@uconn.edu (A.G.M.); Gregory.norris@uconn.edu (G.H.N.); christopher.blesso@uconn.edu (C.N.B.); 2 Department of Nutrition, Universidad Autonoma de Sinaloa, Culiacán, Sinaloa 80019, Mexico; marveji@hotmail.com

**Keywords:** *Moringa oleifera*, hepatic steatosis, inflammation, lipid accumulation, gene expression, guinea pigs

## Abstract

To investigate the mechanisms by which *Moringa oleifera* leaves (ML) modulate hepatic lipids, guinea pigs were allocated to either control (0% ML), 10% Low Moringa (LM) or 15% High Moringa (HM) diets with 0.25% dietary cholesterol to induce hepatic steatosis. After 6 weeks, guinea pigs were sacrificed and liver and plasma were collected to determine plasma lipids, hepatic lipids, cytokines and the expression of genes involved in hepatic cholesterol (CH) and triglyceride (TG) metabolism. There were no differences in plasma lipids among groups. A dose-response effect of ML was observed in hepatic lipids (CH and TG) with the lowest concentrations in the HM group (*p* < 0.001), consistent with histological evaluation of lipid droplets. Hepatic gene expression of diglyceride acyltransferase-2 and peroxisome proliferator activated receptor-γ, as well as protein concentrations interleukin (IL)-1β and interferon-γ, were lowest in the HM group (*p* < 0.005). Hepatic gene expression of cluster of differentiation-68 and sterol regulatory element binding protein-1c were 60% lower in both the LM and HM groups compared to controls (*p* < 0.01). This study demonstrates that ML may prevent hepatic steatosis by affecting gene expression related to hepatic lipids synthesis resulting in lower concentrations of cholesterol and triglycerides and reduced inflammation in the liver.

## 1. Introduction

Non-alcoholic fatty liver disease (NAFLD) is characterized by a variety of liver conditions ranging from hepatic steatosis, to non-alcoholic steatohepatitis (NASH) [1] which, if untreated, can lead to further complications such as hepatic fibrosis, cirrhosis or cancer, and even death [2,3]. There are several dietary interventions that can be used to ameliorate hepatic lipid accumulation, oxidative stress and inflammation associated with NAFLD and NASH [4,5] including the use of foods that are rich in antioxidants, among which polyphenols and carotenoids play a predominate role [6,7].

*Moringa oleifera* Lam is a softwood tree original from the Himalayas and Northern India, which has been investigated for its uses in human health [8]. Many parts of this plant (leaves, immature pods, flowers and fruits) are edible and are used for their nutritional content in many countries [9]. The leaves of the Moringa, the most utilized part of the plant, are characterized by having a great number of bioactive compounds including vitamins, carotenoids, polyphenols (phenolic acids and flavonoids) [10]. For this reason, Moringa leaves (ML) have been used to treat a number of conditions including insulin resistance, cardiovascular disease, hepatic steatosis, cancer and others [11].

ML have also been shown to have hepatoprotective activity and decrease the plasma lipids of rats fed a high-fat diet [12,13]. The aqueous extract of Moringa leaves has been reported to regulate glucose metabolism in diabetic rats [14,15]. In addition, ML have been found to have a cardioprotective role in isoproterenol-induced myocardial infarction by affecting the activities of several enzymes associated with oxidation [16].

Flavonoids and saponins present in ML are reported to increase HDL (high density lipoprotein) cholesterol (HDL-C) and to lower LDL (low density lipoprotein) cholesterol (LDL-C) and very low-density lipoprotein (VLDL) cholesterol in hypercholesterolemic rats [17]. It appears that flavonoids and saponins lower cholesterol absorption by the inhibition of cholesterol micellar solubility [18]. Thus, both of these bioactive compounds found in the aqueous extract have been reported to have hypolipidemic effects [9,10].

Several bioactive compounds including nitrile, mustard oil glycosides and thiocarbamate present in ML have been shown to stabilize blood pressure [19]. In addition, aqueous extracts of leaves, fruit and seeds of Moringa have antioxidant capacities [20], possibly due to the presence of quercetin and kaempferol in Moringa, as they have been reported to have strong antioxidant activity on hepatocyte growth factor-induced oxidation [21]. The liver protection of Moringa leaves has been found in other reports. Ethanolic extracts of ML have shown a protective effect against antitubercular drug-induced liver damage in rats [22]. Further, histological examinations confirmed a decrease in hepatic damages induced by drugs [23]. ML also showed the ability to reduce carbon tetrachloride-induced liver fibrosis and control the rise of serum aminotransferase activities and globulin level [24] and to protect liver exposed to ionizing radiation by increasing antioxidant enzymes and inhibiting nuclear factor κB (NF-κB) translocation to the nucleus [25].

Phenolic acids, including chlorogenic acid (CGA), from ML have been shown to have antioxidant, anti-inflammatory and anti-hyperglycemic properties [26]. However, the specific mechanisms associated with reduction of hepatic lipids have not been fully elucidated.

The present study was conducted to investigate the effect of ML in a guinea pig model of hepatic steatosis. Our hypothesis was that ML would prevent hepatic steatosis induced by a high cholesterol diet by modulating the gene expression of regulators of hepatic cholesterol and triglyceride homeostasis through the action of bioactive compounds present in ML.

## 2. Results

### 2.1. Food Intake and Body and Liver Weight

No differences in food intake, final body weight or liver weight were found among groups during the intervention. Food intake was 24.0 ± 2.2, 20.7 ± 7.8 and 23.6 ± 2.9 g/day for the control, LM and HM groups (*p* > 0.05). Final body weights for guinea pigs were: 535 ± 44, 543 ± 55 and 507 ± 43 g, respectively (*p* > 0.05).

### 2.2. Plasma Lipids, Glucose and Liver Enzymes

There were no differences in plasma total cholesterol, VLDL, LDL, HDL, triglycerides, glucose or insulin among groups. Both Moringa groups had lower activity of ALT than the control group, while no difference in AST between groups was observed (*p* < 0.05) (Table 1).

### 2.3. Lipoprotein Size and Subfractions

The NMR method reports the concentration of lipoproteins in plasma and the size [27]. Total VLDL, total LDL and their subfractions (large, medium, and small) did not differ between groups at the end of the intervention. Although total HDL, large and small HDL were not affected, medium HDL was lower in both groups of Moringa compared to the control group (*p* < 0.05) (Table 2). There were no differences in the VLDL, LDL and HDL size between groups.

### 2.4. LCAT and CETP Activity

**Figure 1:**
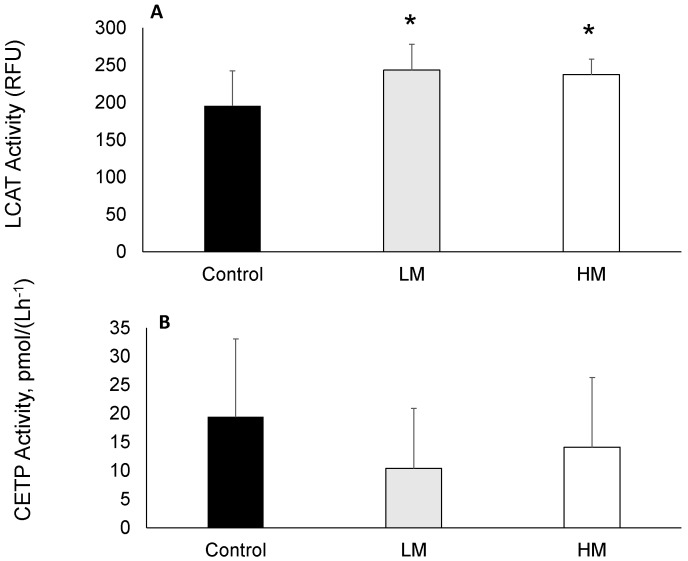
(**A**) Lecithin cholesterol acyltransferase (LCAT) and (**B**) Cholesterol ester transfer protein (CETP) activities of guinea pigs fed control, Low Moringa (LM; 10 g/100 g) and High Moringa (HM; 15 g/100 g) diets. * indicates significantly different from control at *p* < 0.01. RFU, Reference Units.

LCAT activity was greater in guinea pigs fed Moringa compared to controls (Figure 1A) while plasma CETP activity was not different among groups (Figure 1B).

### 2.5. Hepatic Lipids

After the extraction of hepatic lipids [28] and measurement [29,30], TC, CE, and TG were lower in group fed the HM diet (*p* < 0.05), while the concentrations of hepatic lipids in the LM group were not different compared with HM or control groups (Table 3).

### 2.6. Liver Inflammation

Hepatic cytokines were measured simultaneously [31]. The hepatic cytokines IL-1β, 1L-10 and IFNγ were lowest in the HM group, intermediate in the LM group and highest in the control group (*p* < 0.05). There were no differences in IL-6, MCP-1 and TNFα cytokines between groups (Table 4).

### 2.7. Histology

**Figure 2:**
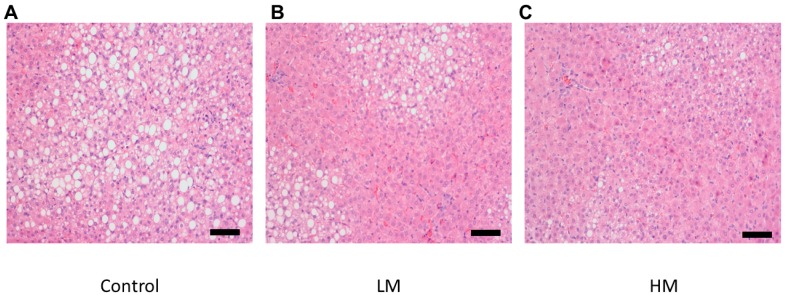
Histological images of hematoxylin and eosin (H&E) (200×) stained hepatic tissue for (**A**) Control, (**B**) Low Moringa (LM; 10%), and (**C**) High Moringa (HM; 15%). Scale bar = 100 μm.

There was a lower degree of steatosis and lipid accumulation in livers from the guinea pigs fed the HM diet compared to the control and the LM group (Figure 2). Liver samples from guinea pigs fed the control diet showed the most lipid accumulation while the LM group exhibited less accumulation than the control in agreement with hepatic cholesterol and TG concentrations.

### 2.8. Hepatic Gene Expression

**Figure 3:**
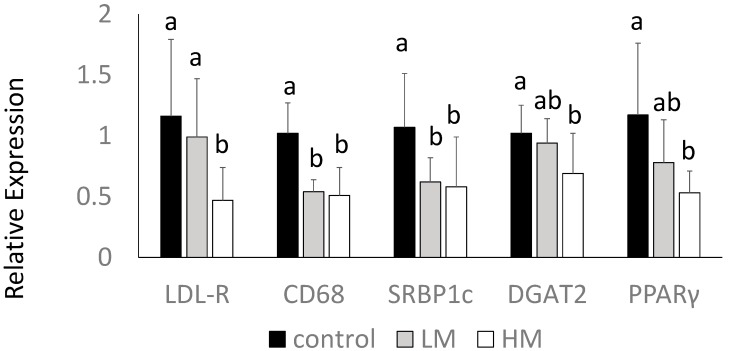
Hepatic gene expression of LDL receptor (*LDL-R*), cluster of differentiation 68 (*CD68*), *SREBP1*, *DGAT2* and *PPARγ* of guinea pigs fed control (**black** bars), Low Moringa (LM; 10%, **gray** bars) or High Moringa (HM; 15%, **white** bars). Different superscripts, a and b denote significantly different at *p* < 0.05.

Interestingly, the HM group had lower levels of LDL receptor (*LDL-R*) gene expression compared to control and LM groups. Guinea pigs fed the ML had lower gene expression of *CD68*, a macrophage marker, and *SREBP1c* mRNA compared to control (*p* < 0.05) (Figure 3). *DGAT2* and *PPARγ* mRNA were lowest in the HM group, intermediate in the LM group, and highest in the control group (*p* < 0.05) (Figure 3). There were no differences in HMG-CoA reductase, *CD36*, *LXR* or *IL-1β* among groups (data not shown).

## 3. Discussion

In this study, we have demonstrated that ML effectively attenuates hepatic steatosis in guinea pigs, leading to the lowering of hepatic lipids and inflammatory markers. We also observed that there was a dose-response with the HM diet having stronger effects on preventing the development of a steatotic phenotype. This could potentially be a consequence of the ingestion of more bioactive compounds present in ML, such as quercetin and CGA, which have been shown to alter gene expression of major regulators of hepatic cholesterol and TG synthesis and uptake [32,33].

### 3.1. Moringa Leaves and Plasma Lipids and Liver Enzymes

We found no differences in food intake or body weight between controls and guinea pigs fed ML, which suggest that ML did not affect weight gain in guinea pigs. Gebregiorgis et al. [34] used different concentrations of ML in sheep and compared to a control diet of Rhodes grass. Sheep consuming the ML had greater body weight gain and nitrogen retention, also suggesting a non-toxic effect.

ALT and AST are enzymes involved in amino acid metabolism and can be used as indicators of liver damage [35]. In this study, a significant reduction was found in ALT activity in both Moringa groups, but not in AST. Other studies have also shown reductions of serum ALT and AST in animals treated with an extract of ML [36]. Pari et al. [22] reported reductions of these liver enzymes with Moringa intake, and a histological evaluation of the liver further documented a recovery from liver damage with ML. These results suggest that ML exert protective effects in liver without inducing toxicity.

The ML used in this study was composed of 19% fiber; therefore, we were expecting to observe decreases in plasma LDL-C due to fiber intake. However, we observed no changes in plasma total or LDL-C among the three dietary groups, suggesting that the fiber present in Moringa did not disrupt micelle formation and the enterohepatic circulation of bile acids as we have observed in guinea pig fed other sources of dietary fiber [37]. Interestingly, guinea pigs consuming the HM had lower expression of LDL-R compared to the other groups. The hepatic LDL-R is the major mechanism by which the body removes LDL-C from plasma [38], and it has been shown that LDL-R [39] and LDL uptake [40] are up-regulated by fiber. In this study, there were no differences in plasma cholesterol among groups, while there was a very significant lowering of hepatic cholesterol in the HM group. This suggests that the decreased expression of LDL-R might have contributed to the lack of effect in lowering plasma LDL-C and to the very significant lowering of hepatic cholesterol.

Consumption of ML, however, resulted in lower concentrations of the medium HDL particles, which have been shown to be less effective in reverse cholesterol transport (RCT), compared with other HDL particle subclasses [41]. A decrease in medium HDL has also been shown to be accompanied by significant increases in HDL-C in the case of low carbohydrate diets [42]. Further suggesting that RCT may have been altered by ML, we observed a 55% increase in LCAT activity, a key regulator of RCT. The major function of LCAT is to facilitate CE formation through the action of Apolipoprotein I (ApoA-I), and it is believed that larger discoidal HDL particles are the preferred substrate for the enzyme [43]. The lipid transfer protein, CETP, is involved in moving CE, TG and phospholipid molecules between HDL and other lipoprotein particles [44]. Thus, LCAT activity influences the efflux efficiency associated with the removal of cellular cholesterol. Thus, although we did not observe changes in atherogenic lipoproteins, the observed changes in these mediators (LCAT, HDL) of RCT suggest ML may also protect against atherosclerosis. Further studies are necessary to confirm this theory.

### 3.2. Moringa Leaves and Hepatic Lipids, Inflammation, and Gene Expression

Previous studies in our laboratory have shown that a cholesterol challenge of 0.25% (*w*/*w*) dietary cholesterol leads to hepatic steatosis in guinea pigs [45]. This accumulation of lipids was observed in the control animals, while the intake of ML resulted in lower concentrations of cholesterol and TG in a dose-dependent manner. CGA is one of the bioactive components present in ML in sufficient amounts to exert hypolipidemic effects [46]. Other investigators have reported that CGA markedly decreases the concentration of total cholesterol and TG in the liver [47], similar to our findings. In addition, Cho et al. [48] reported that CGA significantly inhibited the activities of fatty acid synthase, 3-hydroxy-3-methylglutaryl CoA reductase, and acyl-CoA cholesterol acyltransferase, while it increased fatty acid β-oxidation and the expression of PPARα in mouse livers compared to a control group fed a high-fat diet. We observed decreases in SREBP-1c expression in guinea pigs fed ML. Lipid biosynthesis is regulated at the transcriptional level by SREBP-1 and -2. SREBP-1c is associated with the synthesis of fatty acids and TG, while SREBP-2 with that of cholesterol [49]. CGA, as has been shown in other studies [32], could have also had an effect in decreasing TG via reductions in SREBP1c.

Excess free cholesterol is toxic to cells and induces pro-inflammatory responses which increase the production of cytokines and chemokines [50]. In our study, the control group had 41% higher concentrations of free cholesterol than the Moringa groups, which was accompanied by higher concentrations of the inflammatory cytokines IL-1β and IFNγ and higher expression of the macrophage marker CD68, suggesting that increases in free cholesterol led to hepatic inflammation [50]. Recently, Waterman et al. [51] evaluated the effect of supplementation of a ML concentrate in high fat diet-induced obese mice for 12 weeks. They also found a reduction of gene expression of pro-inflammatory markers, IL-6 and IL-1β in the liver and ileum tissues in mice treated with ML compared to control. CGA may contribute to such effects, as it has shown to suppress the transcription of inflammatory cytokines and inhibit activation of the NF-κB signaling pathway [52]. Histological evaluation of livers also demonstrated less lipid droplet accumulation in the ML groups, in total agreement with hepatic lipids and inflammatory makers.

The decreases in hepatic TG can also be associated with the lower expression of DGAT2. DGAT2, is a key microsomal enzyme involved in TG biosynthesis, and its function is to acylates diacylglycerol at the sn-3 position, using fatty acyl CoAs [53]. Quercetin, a flavonoid, has been shown to reduce TG synthesis in Caco-2 cells, in part via the reduction of DGAT2 activity [54]. Since ML have significant concentrations of quercetin [46], we can speculate that this flavonoid might have been involved in the decreased expression of DGAT2, further contributing to the observed lower concentrations of TG in the guinea pigs fed the ML.

We conclude that ML regulate key genes involved in lipid synthesis, resulting in lower concentrations of lipids and inflammatory cytokines in the liver, demonstrating the effectiveness of ML in reducing fatty liver in cholesterol-fed guinea pigs. Further studies are needed to examine the effects of ML on liver health in humans.

## 4. Materials and Methods

### 4.1. Experimental Design

Male Hartley guinea pigs (*n* = 8 per group) were allocated into three groups: Control, Low Moringa (LM) (10 g/100 g), and High Moringa (HM) (15 g/100 g) for 6 weeks. This amount of Moringa was chosen based on 20 g of food consumed by guinea pigs per day to have 2 or 3.5 g or Moringa per day similar to human studies (Vergara-Jimenez, personal communication). At the end of the 6 weeks, guinea pigs were killed by exsanguination following heart puncture and blood was centrifuged immediately (2000× *g*) to separate plasma. Liver tissue was also collected, snap-frozen and stored at −80 °C until further analysis. All animal protocols were approved by the Institutional Animal Care and Use Committee (Assurance A3124-01) at the University of Connecticut (Protocol #A16-003) on 1 March 2016. Moringa leaves were a gift from Scientech from Health International (Mexico City, Mexico). Macronutrient concentration of the diet was adjusted to account for the inclusion of Moringa in the diet. Moringa composition is presented in (Table 5). A detailed description of the diets is presented in (Table 6).

### 4.2. Plasma Lipids, Glucose and Liver Enzymes

The concentrations of glucose, alanine aminotransferase (ALT), aspartate aminotransferase (AST) and plasma lipids, including total cholesterol (TC), LDL-C, HDL-C and triglycerides (TG) were measured by using Cobas C 111 Analyzer (Roche Diagnostics, Indianapolis, IN, USA).

### 4.3. Lipoprotein Subfraction and Size

The concentrations of large, medium and small VLDL, LDL and HDL particles were measured by nuclear magnetic resonance (NMR). NMR spectroscopy was performed on a 400-MHz NMR analyzer (Bruker Biospin, LipoScience Inc., Raleigh, NC, USA), as described previously [27].

### 4.4. Lecithin Cholesterol Acyl Transferase (LCAT) and Choleterol Ester Protein (CETP) Acitivities

The activity of LCAT was evaluated by use of a commercially available fluorometric assay kit (Cell Biolabs Inc., San Diego, CA, USA). The relative LCAT activity was assessed by the strength of the fluorescence signal following an incubation period. CETP activity was measured by use of a commercially available assay kit (BioVision Inc., Milpitas, CA, USA). The decrease in fluorescence intensity over time was used to calculate CETP activity after incubation of plasma with a self-quenching donor molecule and an acceptor molecule.

### 4.5. Hepatic Lipids

Hepatic tissue (100 mg) from each sample was homogenized with 200 μL of chloroform/isopropanol/MP40 (7/11/1). The mixtures were centrifuged and the supernatant dried at 50 °C overnight. The quantification for TC and free cholesterol (FC) was done with enzymatic assays kits (Wako Diagnostics, Mountain View, CA, USA) [28]. Esterified cholesterol (CE) was calculated manually by subtracting FC from TC. Liver TG were extracted (100 mg of tissue) with chloroform:methanol (2:1), dried under nitrogen at 60 °C and solubilized in 1% Triton X-100. The solubilized lipid isolates were analyzed by enzymatic methods (Wako Diagnostics, Mountain View, CA, USA), as previously described [29].

### 4.6. Histologic Evaluation

Small pieces taken from the same liver section were immersed in 10% buffered formalin. Formalin-fixed livers were paraffin embedded and sections measuring 3–5 μm were stained with hematoxylin. The stained tissue sections were viewed under bright field microscopy at 200× magnification. An AxioCam ICc3 camera (Zeiss, Thornwood, NY, USA) was used to take the pictures.

### 4.7. Inflammatory Cytokine Concentration in Liver

Tissue total protein was extracted and protein concentration of the lysates was evaluated by use of the bicinchoninicc acid assay rotein Assay Kit (Cell Signaling Technologies, Inc., Beverly, MA, USA). Using the same concentration of protein for all samples, the following cytokines were measured using Luminex technology (Luminex 200 System, Austin, TX, USA) with the MILLIPLEX MAP Rat Cytokine Immunoassay kit (Millipore corporation, Charles, MO, USA): interleukin (IL)-1β, IL-6, IL-10, interferon (IFN)-γ, monocyte chemotactic protein 1 (MCP-1), and tumor necrosis factor (TNF)-α, as previously described [30].

### 4.8. Gene Expression

Primer design: The National Center for Biotechnology Information website was used to design the specific primers of *Cavia porcellus*. The following genes involved in lipid metabolism and inflammation were analyzed: LDL receptor (*LDL-R*), cluster of differentiation-68 (*CD68*); HMG-CoA reductase (*HMG-CoA-R*); peroxisome proliferator-activated receptor (*PPARγ*), cluster of differentiation-36 (*CD36*); diacyl glycerol acyltransferase-2 (*DGAT-2*), Primer sequences are available upon request.

### 4.9. RNA Isolation, cDNA Synthesis and Real-Time qRT-PCR

RNA was extracted with TRIzol (Thermo Fisher Scientific, Pittsburgh, PA, USA) after liver tissues were homogenized. RNA was reverse transcribed to cDNA and then amplified and quantified by real-time quantitative polymerase chain reaction using a Bio-Rad CFX96 Real-Time system (BioRad, Hercules, CA, USA), as described [31]. Glyceraldehyde 3-phosphate dehydrogenase (GAPDH) expression was used in normalization using the 2^−ΔΔ*C*t^ method.

### 4.10. Statistical Analysis

Differences among groups were analyzed by one-way ANOVA and Tukey post hoc analysis. *p* < 0.05 was considered to be significant. All analyses were conducted on SPSS for Windows, Version 20 (IBM Corp., Armonk, NY, USA). All data are presented as mean ± Standard Deviation (SD).

## Figures and Tables

**Table 1:** Plasma Lipid profile, glucose, insulin and liver enzymes of guinea pigs fed a hypercholesterolemic diet with no Moringa (control), Low (10%), or High Moringa (15%) ^1^.

Parameter	Control	Low Moringa	High Moringa
Total cholesterol (mg/dL)	294.6 ± 167.0 ^a^	192.9 ± 84.7 ^a^	251.6 ± 70.3 ^a^
VLDL cholesterol (mg/dL)	11.0 ± 2.9 ^a^	10.6 ± 5.0 ^a^	11.9 ± 4.0 ^a^
LDL cholesterol (mg/dL)	244.5 ± 166.4 ^a^	155.0 ± 73.9 ^a^	187.7 ± 73.4 ^a^
HDL cholesterol (mg/dL)	39.1 ± 28.3 ^a^	27.4 ± 24.2 ^a^	52.0 ± 30.3 ^a^
Triglycerides (mg/dL)	55.9 ± 13.9 ^a^	53.6 ± 24.7 ^a^	59.5 ± 18.9 ^a^
Glucose (mg/dL)	158.4 ± 16.5 ^a^	154.4 ± 23.4 ^a^	164.6 ± 18.9 ^a^
ALT (IU/L)	239.3 ± 144.6 ^a^	109.4 ± 43.7 ^b^	141.9 ± 73.6 ^b^
AST (IU/L)	552.9 ± 479.4 ^a^	276.9 ± 146.5 ^a^	299.4 ± 195.1 ^a^

^1^ Values are means ± standard deviation *n* = 8. Values in the same row with different superscripts a and b are significantly different at *p* < 0.05. LDL, low-density lipoprotein; HDL, high-density lipoprotein; VLDL, very low-density lipoprotein; ALT, alanine aminotransferase; and AST, aspartate amino transferase.

**Table 2:** Lipoprotein subfractions in guinea pigs fed a hypercholesterolemic diet with no Moringa (control), Low (10%), or High Moringa (15%) ^1^.

Parameter	Control	Low Moringa	High Moringa
Total VLDL (nmol/L)	55.2 ±1 28.1 ^a^	45.4 ± 21.4 ^a^	61.5 ± 1.7 ^a^
Large VLDL (60–100 nm)	1.7 ± 1.3 ^a^	1.1 ± 0.9 ^a^	1.1 ± 0.9 ^a^
Medium VLDL (40–60 nm)	14.3 ± 7.0 ^a^	13.7 ± 7.6 ^a^	16.7 ± 7.2 ^a^
Small VLDL (30–40 nm)	39.1 ± 21.2 ^a^	30.6 ± 14.0 ^a^	16.7 ± 7.2 ^a^
Total LDL (nmol/L)	531 ± 219 ^a^	563 ± 173 ^a^	709 ± 284 ^a^
IDL (nmol/L)	63 ± 31 ^a^	80 ± 33 ^a^	107 ± 52 ^a^
Large LDL (23–30 nm)	148 ± 61 ^a^	102 ± 46 ^a^	104 ± 30 ^a^
Small LDL (18–23 mn)	320 ± 160 ^a^	382 ± 123 ^a^	498 ± 252 ^a^
Total HDL (µmol/L)	1.5±0.6 ^a^	0.9 ± 0.7 ^a^	1.5 ± 0.5
Large HDL (10–13 nm)	0.19±0.13 ^a^	0.14±0.09 ^a^	0.21±0.08 ^a^
Medium HDL (8.2–10)	0.56± 0.55 ^a^	0.11±0.21 ^b^	0.21±0.13 ^b^
Small HDL (7.3–8.2)	0.8±0.6 ^a^	0.6±0.7 ^a^	1.0±0.5 ^a^
VLDL size (nm)	51.0 ± 5.7 ^a^	46.4 ± 5.5 ^a^	47.4 ± 5.5
LDL size (nm)	20.8 ± 0.6 ^a^	20.3 ± 0.5 ^a^	20.3 ± 0.7 ^a^
HDL size (nm)	9.5 ± 1.0 ^a^	10.1 ± 0.7 ^a^	9.7 ± 0.5 ^a^

^1^ Values are means ± SD, *n* = 8; Means in the same row with different superscripts a and b differ at *p* < 0.05. IDL, intermediate density lipoprotein.

**Table 3:** Concentration of hepatic total cholesterol, free cholesterol, cholesteryl ester and inflammatory cytokines of guinea pigs fed a hypercholesterolemic diet with no Moringa (control), Low (10%), or High Moringa (15%) ^1^.

Parameter	Control	Low Moringa	High Moringa
Total Cholesterol (mmol/g)	28.1 ± 7.2 ^a^	23.3 ± 4.1 ^a,b^	16.0 ± 7.8 ^b^
Free Cholesterol (mmol/g)	11.6 ± 2.3 ^a^	8.3 ± 2.6 ^b^	6.5 ± 4.2 ^b^
Esterified cholesterol (mmol/g)	16.3 ± 5.4 ^a^	15,0 ± 2.8 ^a,b^	9.8 ± 4.7 ^b^
Triglycerides (mmol/g)	57.7 ± 11.5 ^a^	48.8 ± 9.9 ^a,b^	34.5 ± 13.0 ^b^

^1^ Values are means ± SD, *n* = 8; Values in the same row with different superscripts a and b are significantly different at *p* < 0.05.

**Table 4:** Hepatic cytokines/chemokines of guinea pigs fed a hypercholesterolemic diet with no Moringa (control), Low (10%), or High Moringa (15%) ^1^.

Parameter	Control	Low Moringa	High Moringa
IL-1β (ng/g)	196.4 ± 49.2 ^a^	180.3 ± 40.9 ^a,b^	143.1 ± 31.5 ^b^
IL-6 (ng/g)	30.1 ± 3.7 ^a^	27.5 ± 4.9 ^a^	37.3 ± 16.4 ^a^
IL-10 (ng/g)	233 ± 54 ^a^	208 ± 42 ^a,b^	160 ± 38 ^b^
IFNγ (ng/g)	194.4 ± 20.5 ^a^	173.4 ± 39.2 ^a,b^	153.4 ± 42.3 ^b^
MCP-1 (ng/g)	11.4 ± 1.1 ^a^	10.3 ± 1.2 ^a^	21.9 ± 21.7 ^a^
TNFα (ng/g)	31.1 ± 4.9 ^a^	27.4 ± 4.4 ^a^	35.6 ± 19.3 ^a^

^1^ Values are means ± SD, *n* = 8; Values in the same row with different superscripts are significantly different at *p* < 0.05 using Tukeys’ test as post-hoc test. IFNγ, Interferon γ; MCP-1, monocyte chemoattractive protein-1.

**Table 5:** Composition of *Moringa oleifera* leaves.

Component	Amount (g/100 g)
Carbohydrate	30
Fiber	44
Protein	5
Fat	3
Moisture	10
Minerals	8

**Table 6:** Composition of experimental diets.

Component	Control		Low Moringa (LM)		High Moringa (HM)	
	g/100 g	% Energy	g/100 g	% Energy	g/100 g	% Energy
Protein ^1^	22	23	20	23	17	23
Carbohydrate	41	41.9	39	41.9	38	41.9
Fat ^2^	15.1	35.1	14.8	35.1	14.7	35.1
Vitamins ^3^	1.1	-	1.1	-	1.0	-
Minerals ^3^	8.1	-	8.1	-	7.8	-
Cellulose	10	-	7	-	5	-
Guar Gum	2.5	-	2.5	-	2.5	-
Cholesterol	0.25	-	0.25	-	0.25	-
Moringa ^4^	0	-	10	-	15	-

^1^ Soybean protein + 0.5 g/100 g Methionine; ^2^ Fat mix was olive oil-palm kernel oil/safflower oil, high in lauric and myristic acids, as previously reported; ^3^ Composition of vitamin and mineral mixes have been previously reported; and ^4^ Moringa leaves were provided by Scientech Health International (Mexico City, Mexico). The composition is 19% fiber, 20% carbohydrate, 20% protein, 3% lipid and 40% water.
